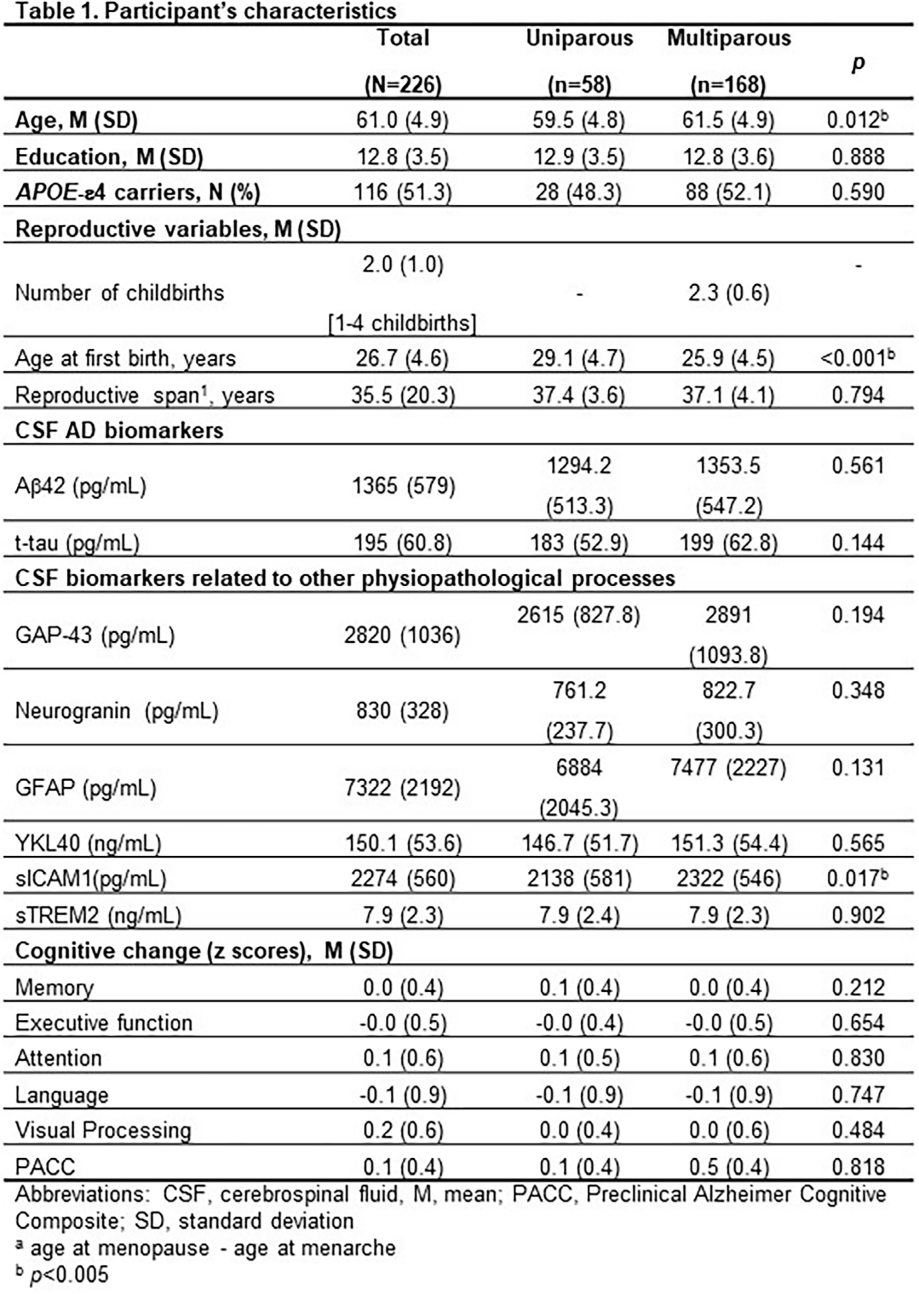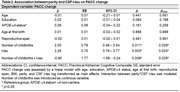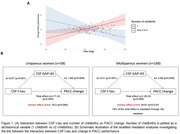# Synaptic dysfunction mediates the association between the CSF t‐tau and PACC change among cognitively unimpaired multiparous women

**DOI:** 10.1002/alz.091314

**Published:** 2025-01-09

**Authors:** Anna Brugulat‐Serrat, Gonzalo Sánchez‐Benavides, David López‐Martos, Armand González Escalante, Marta Milà‐Alomà, Mahnaz Shekari, Nicholas J. Ashton, Gwendlyn Kollmorgen, Raffaele Cacciaglia, Oriol Grau‐Rivera, Clara Quijano‐Rubio, Henrik Zetterberg, Kaj Blennow, Marc Suárez‐Calvet, Erin E. Sundermann, Juan Domingo Gispert, ALFA study

**Affiliations:** ^1^ IMIM (Hospital del Mar Medical Research Institute), Barcelona Spain; ^2^ Global Brain Health Institute, San Francisco, CA USA; ^3^ Centro de Investigación Biomédica en Red de Fragilidad y Envejecimiento Saludable (CIBERFES), Instituto de Salud Carlos III, Madrid Spain; ^4^ Barcelonaβeta Brain Research Center (BBRC), Pasqual Maragall Foundation, Barcelona Spain; ^5^ Barcelonaβeta Brain Research Center (BBRC), Barcelona Spain; ^6^ Centro de Investigación Biomédica en Red de Fragilidad y Envejecimiento Saludable (CIBERFES), Madrid Spain; ^7^ Universitat Pompeu Fabra, Barcelona Spain; ^8^ Department of Radiology and Biomedical Imaging, University of California, San Francisco, San Francisco, CA USA; ^9^ Department of Psychiatry and Neurochemistry, Institute of Neuroscience and Physiology, University of Gothenburg, Mölndal, Gothenburg Sweden; ^10^ Wallenberg Centre for Molecular and Translational Medicine, University of Gothenburg, Gothenburg Sweden; ^11^ King’s College London, Institute of Psychiatry, Psychology & Neuroscience, Maurice Wohl Clinical Neuroscience Institute, London UK; ^12^ NIHR Biomedical Research Centre for Mental Health and Biomedical Research Unit for Dementia at South London and Maudsley, NHS Foundation, London UK; ^13^ Roche Diagnostics GmbH, Penzberg Germany; ^14^ Hospital del Mar Medical Research Institute (IMIM), Barcelona Spain; ^15^ Servei de Neurologia, Hospital del Mar, Barcelona Spain; ^16^ Centro de Investigación Biomédica en Red de Fragilidad y Envejecimiento Saludable (CIBERFES), 28089, Madrid Spain; ^17^ Roche Diagnostics International Ltd., Rotkreuz Switzerland; ^18^ Department of Neurodegenerative Disease, UCL Queen Square Institute of Neurology, University College London, London, ‐ UK; ^19^ UK Dementia Research Institute, University College London, London UK; ^20^ Department of Psychiatry and Neurochemistry, Institute of Neuroscience and Physiology, The Sahlgrenska Academy, University of Gothenburg, Mölndal, Gothenburg Sweden; ^21^ Department of Psychiatry and Neurochemistry, Institute of Neuroscience and Physiology, The Sahlgrenska Academy at the University of Gothenburg, Mölndal Sweden; ^22^ Department of Psychiatry and Neurochemistry, Institute of Neuroscience and Physiology, The Sahlgrenska Academy, University of Gothenburg, Mölndal Sweden; ^23^ Clinical Neurochemistry Laboratory, Sahlgrenska University Hospital, Mölndal Sweden; ^24^ Hospital del Mar Research Institute (IMIM), Barcelona Spain; ^25^ BarcelonaBeta Brain Research Center (BBRC), Pasqual Maragall Foundation, Barcelona Spain; ^26^ University of California, San Diego, La Jolla, CA USA; ^27^ Centro de Investigación Biomédica en Red de Bioingeniería, Biomateriales y Nanomedicina (CIBER‐BBN), Madrid Spain

## Abstract

**Background:**

CSF t‐tau is considered a marker of neuronal injury in AD and strongly correlates with cognitive impairment. Evidence suggests that women accumulate more tau pathology early in AD than men. However, how pregnancy influences this relationship is unclear. We explored association between the number of childbirths and t‐tau and the interaction of these factors on cognitive change in cognitively unimpaired (CU), middle‐aged women and tested whether late‐life biological pathways mediate these associations.

**Method:**

We included 226 CU postmenopausal parous women and 144 men with children from the ALFA+ cohort (Table 1). As late‐life biological pathways we analyzed neuroinflammation (YKL 40, GFAP, and sTREM2), synaptic dysfunction (GAP‐43 and Neurogranin), and vascular dysregulation (sICAM1). All CSF biomarkers were measured using the NeuroToolKit and Elecsys^®^ immunoassays (both Roche Diagnostics International Ltd), except GAP‐43 (ELISA). Cognitive change (3‐year follow‐up) was measured with a PACC‐like composite and individual cognitive domains. Independent linear models with t‐tau and cognitive change as dependent variables were constructed with age, education, APOE‐ε4, reproductive span, and age at first birth as covariates. We replicated the same models, adding Aβ42 as a covariate. Interaction terms between the number of childbirths and biomarkers were modeled. Causal mediation model stratified by number of childbirths was conducted to test whether late‐life biological pathways mediates t‐tau and cognitive change association.

**Result:**

More childbirths were positively associated with higher CSF t‐tau. Number of childbirths interacted with CSF t‐tau to predict a steeper decline in PACC performance (Table 2, Figure 1A) with GAP‐43 levels mediating this relationship (Figure 1B). These results remained significant after covarying by CSF Aβ42. No significant associations among men were found.

**Conclusion:**

A greater number of childbirths are linked to higher CSF t‐tau levels in CU middle‐aged women with a more deleterious effect on the change of PACC performance. This association was partially mediated by the effects of CSF GAP‐43, suggesting synaptic dysfunction in multiparous women in the context of early cognitive deterioration. These findings contribute to our understanding of the higher risk for AD observed in CU multiparous women, who show higher susceptibility and more adverse downstream consequences of tau pathology.